# Enterocyte trafficking proteins and the pathophysiological consequences of their sortopathies

**DOI:** 10.1186/s12876-026-05205-6

**Published:** 2026-08-12

**Authors:** Clenie Isingizwe, Gaudence Ndinganire, Pelagie Izabayo, Abdullateef Isiaka Alagbonsi

**Affiliations:** Department of Physiology, School of Medicine and Pharmacy, College of Medicine and Health Sciences, University of Rwanda, Huye, Rwanda

**Keywords:** Enterocyte trafficking, Protein sorting, Sortopathies, Trafficking proteins

## Abstract

**Objective:**

We synthesised current evidence on enterocyte trafficking proteins and elucidated the molecular mechanisms by which their dysfunction contributes to intestinal disease.

**Methods:**

Search was conducted in PubMed/MEDLINE, Web of Science, and Wiley Online Library for studies published between January 2000 and March 2026, following PRISMA 2020 guidelines. Studies involving enterocyte trafficking proteins were included based on predefined PICO criteria.

**Results:**

Thirty-six studies were included, identifying key trafficking regulators, including Rab8a, Rab11a, MYO5B, MYO1A, MYO6, syntaxin 3, MUNC18-2, and SNX27, as essential for enterocyte protein sorting and polarity. Six major mechanisms of dysfunction were observed, involving defects in protein maturation, vesicle formation, Rab-mediated trafficking, SNARE-dependent fusion, motor-driven transport, and endosomal recycling. These disruptions converge into three main pathological processes: impaired apical delivery, defective endocytic retrieval, and failed exocytic fusion, ultimately leading to severe congenital enteropathies such as microvillus inclusion disease, chylomicron retention disease, and STXBP2-associated enteropathy.

**Conclusion:**

Enterocyte trafficking proteins are fundamental determinants of epithelial polarity and intestinal function. Their dysfunction drives a spectrum of severe intestinal disorders through converging mechanisms of protein mislocalisation and barrier disruption. Advancing diagnostic approaches and developing targeted therapies aimed at restoring intracellular trafficking pathways represent critical priorities for improving patient outcomes.

**Graphical Abstract:**

**Figure:**
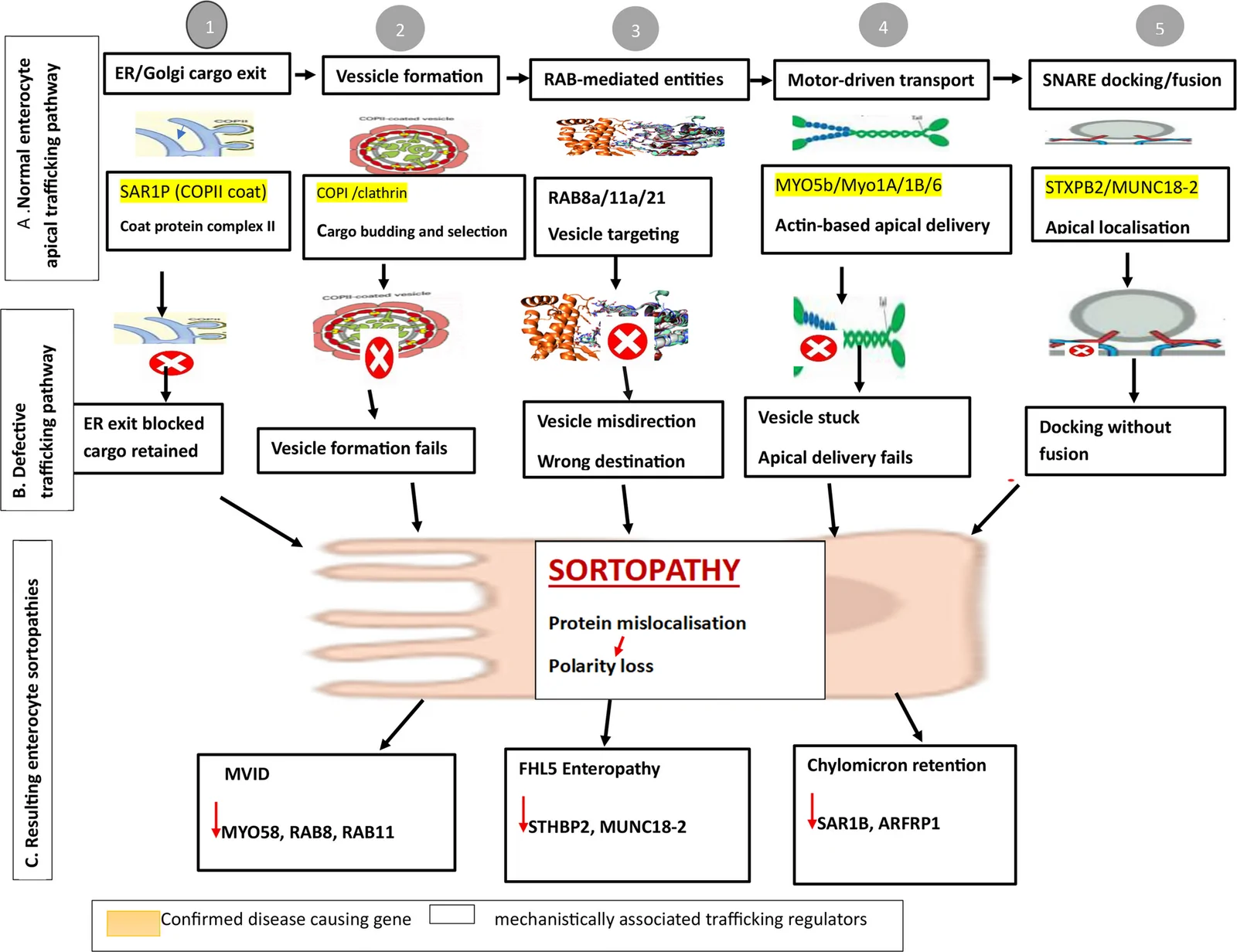

**Supplementary Information:**

The online version contains supplementary material available at https://doi.org/10.1186/s12876-026-05205-6.

## Background

The intestinal epithelium is a dynamic, highly specialised barrier that is in charge of host defence, electrolyte balance, and nutritional absorption [46]. Enterocytes, the polarised epithelial cells with unique apical and basolateral membrane domains that line the small intestine, are essential to this system [49]. Directional transport mechanisms, such as the intake of glucose, amino acids, lipids, and ions, depend on this polarity [58]. Tightly controlled intracellular trafficking pathways that guarantee the proper delivery, recycling, and degradation of membrane proteins are essential for maintaining enterocyte polarity [38, 46, 64]. The apical brush border membrane needs to be precisely targeted by a variety of transporters and channels. Endoplasmic reticulum (ER) quality control systems, vesicular coat proteins, small guanosine triphosphate proteins (GTPases), with SNARE complexes, adaptor proteins, and cytoskeletal motor proteins are all part of the complex protein sorting machinery that mediates this process [15, 34, 54]. These elements maintain enterocyte shape and absorptive ability by coordinating the synthesis, folding, trafficking, docking, and recycling of membrane proteins.

Among the most critical trafficking regulators in enterocytes are the Rab family of small GTPases. Rab8a and Rab11a, for example, orchestrate apical membrane delivery and recycling: Rab8a is required for proper localisation of apical proteins while Rab11a controls the return of internalized cargo to the brush border [29, 50, 52]. Their functional coordination involves Rab11‑FIP2, which links Rab11a to the motor protein MYO5B, thereby stabilising recycling vesicles [38] and movement along the actin cytoskeleton [51]. Likewise, Rab21 participates in early endosomal trafficking and contributes to intestinal epithelium maintenance [41]. In addition, retromer components such as SNX27 facilitate endosome‑to‑plasma membrane trafficking of signalling receptors and transporters, highlighting the importance of endosomal recycling in enterocyte physiology [33, 54, 57].

Motor proteins also play indispensable roles. MYO5B, an actin‑based motor protein, is a central player in apical trafficking; its absence leads to the accumulation of Rab11‑Rab8‑positive vesicles and defective exocytosis of cargo to the brush border [48, 60, 61]. MYO1B, in collaboration with MYO5B and SNX27, directs the apical targeting of the membrane mucin MUC17, which forms the protective glycocalyx [5, 26]. MYO1A is required for cystic fibrosis transmembrane regulator (CFTR) brush border membrane trafficking and ion transport [4, 31], whereas MYO6 functions in endocytic uptake and maintenance of brush border ultrastructure [8, 21]. The interplay between opposing motors (MYO1A and MYO6) fine‑tunes membrane tension and vesicle dynamics, as revealed by double‑knockout studies [31, 37]. When these trafficking routes are disrupted, important transport proteins mislocalise or degrade, impairing epithelial function. These flaws are becoming more widely acknowledged as being essential to a class of diseases associated with enterocyte sortopathies, where disease phenotypes that were previously thought to be solely caused by transporter dysfunction are driven by abnormalities in intracellular protein sorting [14, 30, 43]. For instance, mutations in trafficking regulators such as MYO5B alter apical recycling pathways, leading to microvillus inclusion disease, which is characterized by loss of epithelial polarity and severe diarrhoea [14]. The disruption of vesicular transport and membrane targeting brought on by anomalies in Rab GTPases, SNARE proteins, and retromer complexes further highlights the importance of intracellular sorting in intestinal physiology [3, 9, 51, 60, 61, 66].

We propose the term "sortopathies" to collectively describe intestinal disorders in which the primary pathological mechanism is defective intracellular protein sorting, specifically, the mislocalisation of apical membrane proteins caused by mutations in non-redundant components of the enterocyte trafficking network, rather than mutations in the cargo proteins themselves. This distinguishes sortopathies from primary transporter defects, where the transporter gene is directly mutated, and from structural enteropathies, where epithelial architecture is disrupted through non-trafficking mechanisms. The term is constructed by analogy with established disease nomenclature, as channelopathies denote disorders of ion channel function [27] and ciliopathies denote disorders of cilia [62], to provide a mechanistically coherent label for a group of severe congenital intestinal disorders, including microvillus inclusion disease, chylomicron retention disease, and FHL5 enteropathy, that share a common pathological basis of trafficking network failure despite involving structurally unrelated proteins.

The majority of research has traditionally concentrated on the functional characteristics of individual transporters rather than the upstream trafficking systems that control their localization, despite the growing understanding of these mechanisms. This has hindered the creation of focused treatments that deal with the underlying reasons of enterocyte malfunction. Thus, a thorough comprehension of the machinery involved in protein sorting and its function in intestinal illness is required. In order to provide a molecular framework connecting intracellular sorting abnormalities to intestinal pathophysiology, this review attempts to compile the most recent data on enterocyte trafficking proteins and their function in disease conditions.

## Methodology

### Study design

This study was designed as a systematic review reported in accordance with the Preferred Reporting Items for Systematic Reviews and Meta-Analyses (PRISMA) 2020 guidelines [45], as adopted in previous systematic reviews [17–20, 24, 42, 43]. The PRISMA checklist that guided the review can be assessed as Supplementary File S1. As this review was mechanistic in nature, synthesising experimental, genetic, and laboratory evidence on intracellular trafficking proteins rather than evaluating clinical interventions or patient outcomes, prospective registration on PROSPERO or a similar platform was not pursued, consistent with the primary intent of PROSPERO for intervention and diagnostic systematic reviews [6].

### Search strategy

A comprehensive literature search was performed in the following electronic databases: PubMed/MEDLINE, Wiley Online Library, and Web of Science. The search covered the period from January 2000 to March 2026. This time frame was selected because the early 2000 s marked a period of major advances in molecular genetics, transgenic and conditional knockout animal models, and live-cell imaging technologies, which enabled mechanistic investigation of intracellular trafficking, epithelial polarity, and protein sorting in intestinal epithelial cells. Consequently, studies published from 2000 onward increasingly focused on the functional and disease-related roles of trafficking proteins, whereas earlier studies primarily established the fundamental biology of trafficking molecules and were used as background references where appropriate. The search strategy combined controlled vocabulary (MeSH terms) and free‑text keywords related to three conceptual domains: (1) enterocyte trafficking proteins (e.g., “enterocyte”, “brush border”, “apical membrane”, “Rab GTPase”, “myosin”, “SNARE”, “retromer”, “sorting nexin”); (2) sortopathies (e.g., “microvillus inclusion disease”, “MVID”, “chylomicron retention disease”, “enteropathy”, “trafficking defect”, “mislocalisation”); and (3) molecular mechanisms (e.g., “protein sorting”, “endocytosis”, “recycling”, “exocytosis”, “polarity”). Boolean operators (AND, OR) were used to combine terms. In addition to database searches, reference lists of included articles and relevant reviews were hand‑searched to identify additional eligible studies. The search strings used to obtain articles from each database are presented in Supplementary File S2.

### Inclusion and exclusion criteria

Pre-defined inclusion and exclusion criteria, guided by the PICO framework, were used to screen articles selected in this systematic review (Table 1).

**Table 1:** Eligibility criteria guided by the PICO framework

Category	Inclusion Criteria	Exclusion Criteria
Population (P)	Studies involving intestinal epithelial cells (enterocytes), including human subjects, animal models (e.g., mice, rats, *Drosophila*), and in vitro systems (e.g., Caco-2 cells, intestinal organoids)	Studies not involving enterocytes or intestinal epithelial models
Intervention/Exposure (I)	Studies investigating enterocyte trafficking proteins (e.g., Rab proteins, myosins, SNAREs, sorting nexins) and related molecular pathways involved in protein sorting, vesicular transport, and epithelial polarity	Studies unrelated to trafficking proteins, membrane transport, or intracellular sorting mechanisms
Comparator (C)	Studies comparing normal vs. defective trafficking mechanisms, wild-type vs. mutant models, or treated vs. untreated conditions (where applicable)	Studies lacking a comparator group or without any form of functional or mechanistic comparison
Outcomes (O)	Data on protein trafficking defects, vesicular transport dynamics, membrane localization, microvillus formation, intracellular inclusions, and associated enteropathies (e.g., microvillus inclusion disease—MVID)	Studies with no relevant outcomes related to trafficking mechanisms or enterocyte function/pathophysiology
Time Frame	Studies published from January 2000 to March 2026	Studies published outside this date range
Study Types	Experimental studies, genetic studies, mechanistic studies, in vivo and in vitro functional assays	Reviews, editorials, commentaries, conference abstracts without primary data
Molecular Targets	Studies involving key trafficking regulators (e.g., MYO5B, Rab11, Rab8, SNX proteins, STX3, MUNC18-2, αSNAP, ARFRP1)	Studies not involving trafficking-related proteins or pathways
Language	Articles published in English	Non-English publications
Accessibility	Full-text articles available	Abstract-only studies or inaccessible full texts
Model Validity	Studies with experimental validation in biological systems	Purely computational or theoretical studies without experimental validation

### Article selection, data extraction, and analysis

All retrieved records were imported into reference management software, and duplicates were removed before screening. Study selection followed a two-stage process: initial title and abstract screening, followed by full-text review based on predefined inclusion and exclusion criteria. Two reviewers independently conducted the screening, with disagreements resolved through discussion or consultation with a third reviewer. From each included study, detailed data were extracted, including the first author and year of publication, study design (in vivo, in vitro, or combined), and model system characteristics (such as animal models, human tissues, organoids, or cell lines). Information on the type of experimental intervention (e.g., gene editing, RNA interference, overexpression, or pharmacological approaches) was collected, along with the specific proteins or trafficking pathways investigated. Additionally, key outcome measures related to epithelial structure and function were recorded, as well as the proposed mechanisms of dysfunction, including defects in vesicle trafficking, apical targeting, recycling pathways, cytoskeletal organisation, and protein degradation processes.

A pre-made and tested standardised data extraction form was used by several reviewers to manually extract all of the data [1, 2, 17–20, 24, 28, 42, 44]. To ensure consistency and accuracy in the synthesis, two reviewers independently assessed each study for eligibility and data extraction. Any disagreements in interpretation or classification were resolved through discussion and consensus, with a third reviewer consulted when necessary. Data extraction was presented as Supplementary file S3.

### Mechanistic categorisation and thematic analysis

The findings were synthesised in a way that reflected the functional consequences of enterocyte trafficking abnormalities as well as their molecular mechanisms. First, data were categorized mechanistically according to the stage of trafficking interruption and the intracellular location. This includes abnormalities in vesicular transport and recycling (endosomal trafficking), terminal apical membrane targeting, and early biosynthetic pathways (endoplasmic reticulum and Golgi processing). These classifications enabled the distinction between downstream effects on epithelial structure and function, including compromised brush border development, and primary sorting machinery deficiencies, such as anomalies in Rab GTPases, motor proteins, and sorting nexins. The results were further contextualised within disease states, distinguishing between pathological situations such as microvillus inclusion disease (MVID), other congenital enteropathies, and normal enterocyte physiology. This made it possible to compare complete trafficking systems with damaged pathways that result in clinical symptoms such as diarrhoea, malabsorption, and problems in epithelial polarity.

The review then mapped mechanistic discoveries to particular cellular processes, such as apical vesicle trafficking, endosomal recycling, and ER-to-Golgi transport. Rab11–Rab8 regulatory networks, MYO5B-dependent motor transport, SNARE-mediated vesicle docking (such as syntaxin 3 and MUNC18-2), and retromer/sorting nexin-mediated cargo sorting were all given special attention. To demonstrate how coordinated trafficking guarantees proper apical membrane protein localization and enterocyte polarity preservation, several interrelated routes were examined. The results of the thematic analysis were combined into the following functional categories: (i) aberrant membrane protein maturation and polarized delivery, (ii) impaired vesicular assembly and cargo sorting mechanism, (iii) disruption of Rab GTPase–regulated intracellular trafficking (iv) defective SNARE-dependent vesicle docking and fusion, (v) dysregulation of cytoskeletal motor-driven transport and sorting complexes, (vi) compromised endosomal recycling and membrane retrieval pathways.

The review also found discrepancies amongst research, such as different phenotypic results from mutations in the same trafficking protein (e.g., MYO5B or Rab11), which may be caused by variations in model systems or compensatory pathways. Lastly, a number of important knowledge gaps were identified, such as the lack of knowledge regarding protein-specific cargo identification, the redundancy of trafficking routes, and the impact of cellular environment and developmental stage on trafficking dynamics. To better understand the pathophysiological effects of enterocyte sortopathies, integrative research involving genetic, molecular, and in vivo methods was stressed.

Within each mechanistic category, studies were organised by experimental system, distinguishing human tissue or patient samples, animal models, and in vitro cell lines to contextualise the nature and translational relevance of each finding. Human studies were considered to provide direct clinical evidence of disease relevance; animal models were considered to establish causal mechanistic evidence through controlled in vivo genetic manipulation; and in vitro cell line studies were considered to define molecular detail requiring further validation. Accordingly, findings from each experimental system were assigned a three-tier translational relevance rating, matching the terminology used in Table 2. It should be noted that this classification represents an author-defined translational relevance rating based on experimental model type, rather than a formal clinical evidence-grading system.

- Level 1 (Human): Human tissue or patient-derived samples, representing the highest translational relevance.
- Level 2 (Animal): Animal models (e.g., mouse knockouts) or patient-derived cells, representing moderate translational relevance.
- Level 3 (Cell lines/Invertebrate): Cell lines or invertebrate models, representing the lowest translational relevance and requiring further validation. These studies were retained because they were the systems in which core, largely conserved trafficking machinery (Rab GTPase-motor interactions, retromer-mediated cargo recycling, SNARE-dependent fusion) was first mechanistically defined, before confirmation of the same mechanisms in intestinal-specific systems. So, their inclusion reflects mechanistic, rather than tissue-specific, relevance.

**Table 2:** Mechanism-specific roles of enterocyte trafficking proteins in sortopathies

Authors (Year)	Experimental Model	Evidence Level (Translational Relevance)	Key Findings	Mechanisms Involved	Translational Gap
1. Aberrant Membrane Protein Maturation and Polarized Delivery					
Knowles et al. [29]	Human + Mouse	Level 1 – Human	MYO5B mutations cause congenital diarrhoea with loss of apical polarity	Defective MYO5B uncouples Rab8a/Rab11a, disrupting apical protein targeting	Partially filled human genetic data confirmed in a mouse model
Yu et al. [65]	Mouse KO	Level 2 – Animal	Loss of apical polarity and brush border defects in MYO5B knockout	MYO5B regulates apical vesicle delivery and membrane polarisation	Human validation limited; mechanism inferred from murine model
Engevik et al. [14]	Mouse model	Level 2 – Animal	Reproduced the full MVID phenotype, including inclusion bodies	Loss of MYO5B impairs apical trafficking and inclusion formation	No direct human tissue mechanistic data; mouse phenotype recapitulates disease
Collaco et al. [10]	Mouse	Level 2 – Animal	Syntaxin 3 knockdown mislocalises apical proteins	Syntaxin 3 regulates apical SNARE-dependent membrane fusion	Human validation absent; role of STX3 inferred from murine intestine
2. Impaired Vesicular Assembly and Cargo Sorting					
Jäverfelt et al. [26]	Mouse	Level 2 – Animal	Cooperative failure of MYO1B/MYO5B/SNX27 disrupts MUC17 glycocalyx targeting	Sorting complexes coordinate apical cargo delivery via actin-based transport	Human validation absent; mechanistic insight from mouse intestinal epithelium
Cox et al. [11]	Mouse	Level 2 – Animal	Endotubin loss causes enterocyte differentiation failure	Endotubin regulates endosomal sorting during enterocyte maturation	No human data; role established only in murine knockout model
Wong et al. [63]	Hamster (ex vivo intestinal enterocyte vesicles)	Level 2 – Animal	Proteomic profiling identified key prechylomicron transport vesicle proteins	Vesicle protein composition determines cargo specificity	Ex vivo hamster enterocyte model; translational relevance to human chylomicron retention unconfirmed
Temkin et al. [57]	HEK293 (Human, embryonic kidney; non-intestinal)	Level 3 – In vitro	SNX27 depletion causes receptor mislocalisation	Endosomal sorting defects impair retromer-mediated cargo distribution	Cell line data only; human intestinal relevance not directly demonstrated
Pons et al. [47]	Caco-2 (Human, colon) + COS-7 (Monkey, kidney; non-intestinal)	Level 3 – In vitro	Enterophilin-1/SNX1 interaction alters EGFR surface expression	SNX1 regulates endosomal cargo sorting and receptor trafficking	In vitro only; physiological relevance in human enterocytes unestablished
3. Disruption of Rab GTPase-Regulated Intracellular Trafficking					
Sato et al. [50]	Mouse KO	Level 2 – Animal	Rab8 KO causes brush border collapse and apical protein mislocalisation	Rab8 GTPase required for epithelial polarity and apical protein delivery	No human Rab8a loss-of-function disease identified; gap in human evidence
Sobajima et al. [52]	Mouse intestine	Level 2 – Animal	Rab11a depletion impairs apical protein localisation	Rab11a directs apical vesicle transport in intestinal epithelium	Human-specific Rab11a enteropathy has not yet been described; animal model only
Carton-Garcia et al. [7]	Mouse KO	Level 2 – Animal	MYO5B KO recapitulates MVID intestinal architecture loss	Rab8-dependent apical protein delivery is disrupted in MYO5B absence	Mouse model — human confirmation available via MYO5B mutation studies
Nassari et al. [41]	Drosophila	Level 3 – Invertebrate	Rab21 loss causes an enteropathy-like phenotype	RAB21 regulates early endosomal trafficking in intestinal epithelium	Invertebrate model only — human Rab21 intestinal role unconfirmed; major gap
Vogel et al. [60, 61]	Human (patient duodenal biopsy) + Caco-2 (Human, colon; genome-edited KO)	Level 1 – Human	Rab11–Rab8 vesicle clustering is disrupted in MVID patient-derived cells	Rab11–Rab8 coordination controls apical trafficking fidelity	Human biopsy evidence with cell-line mechanistic support; largest cohort still limited
Thoeni et al. [59]	Caco-2 (Human, colon; siRNA knockdown)	Level 3 – In vitro	MYO5B loss impairs Rab11-dependent recycling endosome dynamics	Rab11 controls endosomal recycling; MYO5B loss arrests this process	Cell line model; mechanistic detail not confirmed in intact human tissue
4. Defective SNARE-Dependent Vesicle Docking and Fusion					
Stepensky et al. [55]	Human (patient duodenal and renal biopsy)	Level 1 – Human	STXBP2 mutations cause persistent membrane trafficking defects in patient epithelial cells	STXBP2/MUNC18-2 mutation impairs SNARE complex assembly and vesicle fusion	Direct human biopsy evidence; mechanistic detail extended by later patient-derived cell/organoid studies
Lechuga et al. [35]	Mouse	Level 2 – Animal	αSNAP loss impairs Paneth cell differentiation and vesicle fusion	αSNAP regulates SNARE complex disassembly and vesicle fusion competence	Mouse data only; human αSNAP enteropathy not characterised
Vogel et al. [60, 61]	Human (patient biopsy/organoid) + Caco-2 (Human, colon; CRISPR KO)	Level 1 – Human	Munc18-2 depletion causes subapical vesicle accumulation and fusion blockade	Munc18-2 is required for apical vesicle docking and SNARE-mediated fusion	Human biopsy and organoid evidence; confirmed at clinical level by FHL5 patient phenotype
Schafer et al. [51]	HeLa (Human, cervical) + MDCK (Canine, kidney); non-intestinal	Level 3 – In vitro	Rab11-FIP2 regulates vesicle movement and docking at the apical membrane	Rab11-FIP2 bridges Rab11a to MYO5B enabling vesicle docking	In vitro mechanistic study; human docking defect inferred from MVID phenotype
5. Dysregulation of Cytoskeletal Motor-Driven Transport and Sorting Complexes					
Knowles et al. [30]	Mouse + Caco-2	Level 3 – In vitro	MYO5B loss causes inclusion body formation and brush border collapse	MYO5B regulates Rab11a-dependent vesicle transport along the actin cytoskeleton	Mouse + cell line; human confirmation via MYO5B mutation genetics
Kravtsov et al. [31]	Mouse	Level 2 – Animal	MYO1A KO impairs CFTR brush border trafficking	Myosin Ia is required for CFTR apical targeting and ion transport	Mouse model only; human MYO1A intestinal disease not yet reported
Jaschke et al. [25]	Mouse	Level 2 – Animal	ARFRP1 KO disrupts chylomicron lipidation and Golgi exit	ARFRP1 GTPase regulates Golgi lipidation of prechylomicron vesicles	Mouse KO — human equivalent is chylomicron retention disease (SAR1B)
Hegan et al. [21]	Mouse	Level 2 – Animal	MYO6 KO disrupts brush border structure and endocytic uptake	Myosin VI maintains brush border architecture and clathrin-mediated endocytosis	Mouse model; human MYO6 intestinal phenotype not described
Hegan et al. [22]	Mouse	Level 2 – Animal	MYO1A/MYO6 double KO causes compounded transport failure	Myosin Ia and VI coordinate opposing membrane trafficking directions	Mouse double KO — no human equivalent identified; gap in translational data
Golin-Bisello et al. [16]	Mouse	Level 2 – Animal	STa/cGMP stimulation increases CFTR apical trafficking via the cytoskeleton	Cytoskeletal motor activity supports secretory CFTR exocytosis	Mouse intestinal loops; human CFTR trafficking partly confirmed in CF studies
Das et al. [13]	Mouse (in vivo Rab8a knockout crypts + intestinal enteroids)	Level 2 – Animal	Rab8a vesicles regulate Wnt delivery at the intestinal stem cell niche	Cytoskeletal trafficking disruption impairs stem cell niche signalling	Mouse in vivo and enteroid data; human intestinal Rab8a-Wnt axis not directly confirmed
Lapierre et al. [34]	HeLa (Human, cervical) + MDCK (Canine, kidney); non-intestinal	Level 3 – In vitro	MYO5B associates with plasma membrane recycling systems	Rab11–MYO5B motor interaction drives apical recycling endosome movement	Original in vitro characterisation; mechanism confirmed in vivo in later studies
Crawley et al. [12]	LS174T-W4 + Caco-2 BBE (Human, colon; intestinal)	Level 3 – In vitro	Protocadherin-based adhesion drives brush border assembly	Actin cytoskeleton and intermicrovillar adhesion are required for microvilli	Cell line model; brush border assembly mechanism conserved in human enterocytes
Stechly et al. [53]	HT-29 (Human, colon; enterocyte-like)	Level 3 – In vitro	Galectin-4 regulates glycoprotein delivery to the brush border	Cytoskeletal transport and lectin-mediated sorting direct apical glycoproteins	In vitro only; human galectin-4 apical sorting role not confirmed in vivo
6. Compromised Endosomal Recycling and Membrane Retrieval Pathways					
Müller et al. [40]	Human	Level 1 – Human	MYO5B mutations identified in MVID patients cause congenital diarrhoea	MYO5B mutation disrupts the apical recycling endosome pathway, the strongest human evidence	Gap filled with direct human genetic and histological evidence
Marchiando et al. [38]	Mouse	Level 2 – Animal	Caveolin-1-dependent occludin endocytosis disrupts tight junctions in vivo	Tight junction internalisation via the endosomal pathway causes barrier dysfunction	Mouse in vivo model; human tight junction endocytosis in IBD is partially confirmed
Cox et al. [11]	Mouse	Level 2 – Animal	Endotubin loss impairs endosomal retrieval during differentiation	Endosomal retrieval pathways required for enterocyte maturation	Mouse only; human endotoxin role in differentiation not established
Steinberg et al. [54]	HeLa (Human, cervical); non-intestinal	Level 3 – In vitro	SNX27–retromer globally regulates glucose and metal ion transporter recycling	Retromer complex directs endosome-to-plasma membrane cargo retrieval	Cell line proteomics: human intestinal retromer deficiency has not been clinically described
Vogel et al. [60, 61]	Human (patient biopsy) + Caco-2 (Human, colon; genome-edited KO)	Level 1 – Human	Rab11–Rab8 vesicle clustering was disrupted, causing recycling failure	Rab11–Rab8 recycling pathway essential for sustained apical membrane composition	Human biopsy evidence with cell-line mechanistic support; intact tissue recycling kinetics not fully resolved
Temkin et al. [57]	HEK293 (Human, embryonic kidney); non-intestinal	Level 3 – In vitro	SNX27 mediates retromer tubule entry for receptor recycling	SNX27–retromer axis controls endosome-to-membrane trafficking of receptors	In vitro mechanistic study; human intestinal SNX27 deficiency not characterised
Stechly et al. [53]	HT-29 (Human, colon; enterocyte-like)	Level 3 – In vitro	Galectin-4 loss impairs brush border glycoprotein recycling	Endosomal recycling impairment causes glycoprotein mislocalisation	Cell line only; human in vivo recycling role of galectin-4 unconfirmed

*Legends*: Level 1: Human tissue/patient samples (highest translational relevance); Level 2: Animal models, including mouse KO, in vivo (moderate translational relevance); Level 3: Cell lines/invertebrate models (mechanistic only). Non-intestinal cell lines (e.g., HeLa, HEK293, COS-7, MDCK) were retained at Level 3 where they established core trafficking mechanisms subsequently confirmed in intestinal-specific systems. So, their translational relevance is mechanistic rather than tissue-specific

### Risk of Bias (RoB) assessment

To evaluate the methodological quality of studies included in this review on enterocyte trafficking proteins and the pathophysiological consequences of their sortopathies, a structured Risk of Bias (RoB) assessment was conducted according to study design. Animal studies were appraised using the SYRCLE Risk of Bias tool, which evaluates domains such as randomization, allocation concealment, blinding, incomplete outcome data, and selective reporting [23]. Human studies were assessed using the Cochrane Risk of Bias 2.0 tool for randomized controlled trials, addressing bias related to the randomization process, deviations from intended interventions, missing data, outcome measurement, and reporting [56]. While observational studies were evaluated using the Newcastle–Ottawa Scale, focusing on selection, comparability, and outcome/exposure assessment [36]. In vitro studies were assessed using a modified Checklist for Reporting In Vitro Studies (CRIS), emphasising experimental design, reproducibility, control conditions, and statistical rigor [32]. All studies were independently assessed by two reviewers, with disagreements resolved through consensus or consultation with a third reviewer. Each study was classified as having low, moderate, or high risk of bias, and findings were summarized in tabular form (Supplementary File S4). Sensitivity analyses were conducted by excluding high-risk studies to evaluate the robustness of the overall conclusions.

## Results

**Fig. 1:**
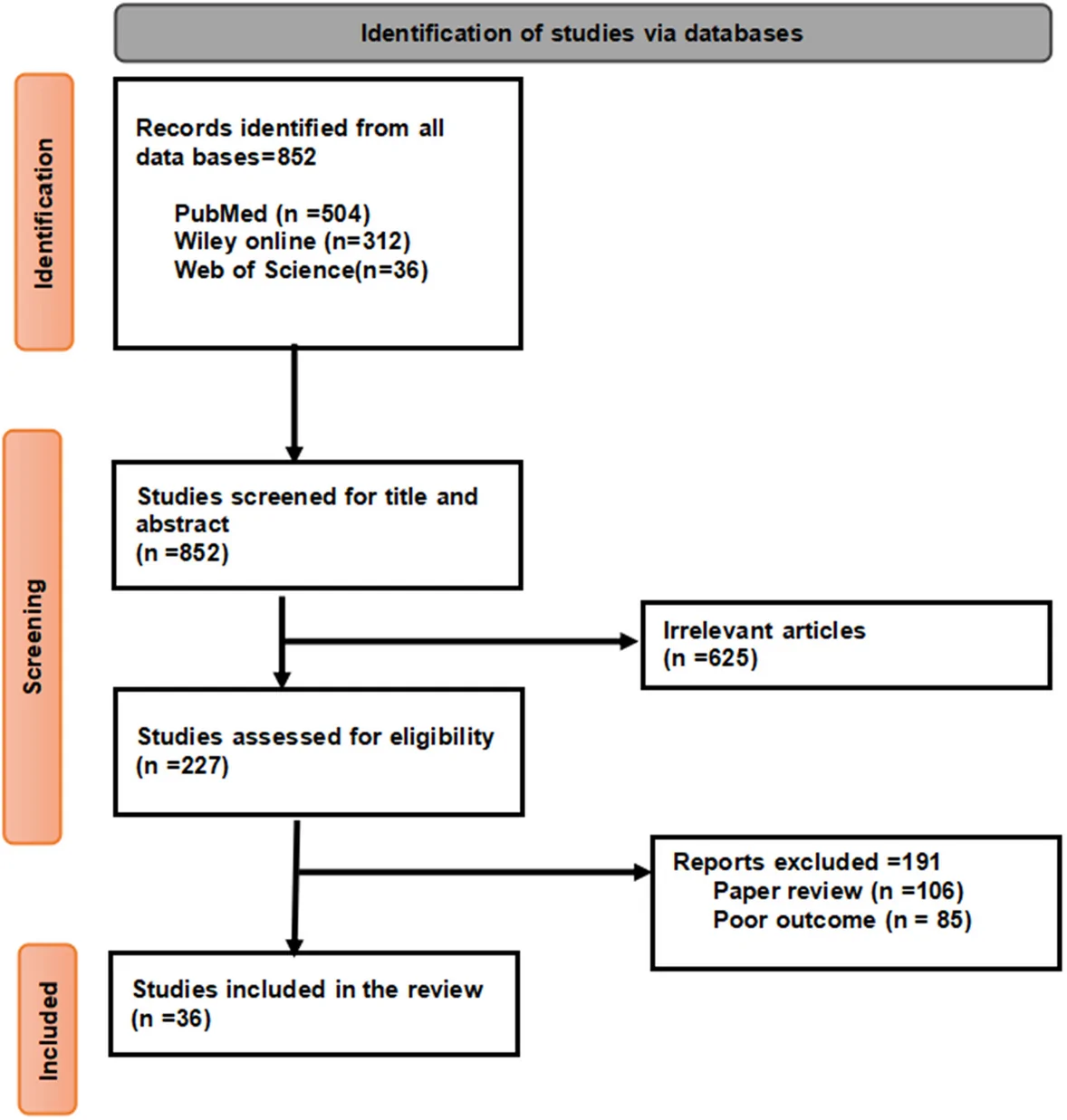
PRISMA flow diagram for included studies

The systematic search identified 852 records from databases, out of which 625 irrelevant or duplicate studies were removed. About 227 full-text articles were screened and assessed for eligibility, out of which 191 were further excluded for specific reasons, and ultimately, 36 studies were included in the review. The study selection process is summarised in the PRISMA flowchart Fig. 1.

### Study characteristics

Across the 36 studies, aberrant membrane protein maturation and polarized delivery (*n* = 4 studies), defective SNARE-dependent vesicle docking and fusion (*n* = 4 studies), impaired vesicular assembly and cargo sorting mechanism (*n* = 5 studies), disruption of Rab GTPase–regulated intracellular trafficking (*n* = 6 studies), compromised endosomal recycling and membrane retrieval pathways (*n* = 7 studies), and dysregulation of cytoskeletal motor-driven transport and sorting complexes (*n* = 10 studies) were the mechanistic categories observed (Table 2). Overall, the evidence base comprised a mixture of experimental designs with differing translational relevance. Human-based studies provided direct clinical evidence but were limited to a small number of well-characterised genetic mutations, principally MYO5B, SAR1B, and STXBP2. Animal models, predominantly mouse knockouts, provided causal mechanistic evidence by demonstrating in vivo consequences of specific protein loss, and in several cases directly phenocopied human disease [7]. In vitro cell line studies contributed molecular mechanistic detail regarding protein interactions and trafficking dynamics, but require human or animal validation to confirm physiological relevance. Findings from invertebrate models such as Drosophila demonstrate evolutionary conservation of trafficking mechanisms, but cannot be directly extrapolated to human intestinal disease [41]. These distinctions are reflected in Table 2, where studies within each mechanistic category are ordered by translational relevance, human evidence first, animal models second, and in vitro data last, and key findings are contextualised by experimental system throughout.

### Synthesis of findings

#### Aberrant membrane protein maturation and polarised delivery

Epithelial protein sorting dysfunction is primarily caused by defective membrane protein maturation and polarised delivery, which includes reduced synthesis, folding, and apical trafficking of important membrane transporters. Several experimental systems have shown that disruption of these mechanisms results in mislocalisation or decreased surface expression of critical epithelial proteins. For instance, in mouse knockout models, the removal of crucial regulatory components modifies the dynamics of membrane protein trafficking [65]. Conserved abnormalities in epithelial transport pathways are further revealed by complementary functional studies in mice and humans [30]. Furthermore, abnormal protein processing directly impairs epithelial function, as demonstrated by decreased CFTR trafficking to the apical membrane [16]. This mechanism is also supported by human genetic mutation investigations, which demonstrate that changes in epithelial transport proteins lead to loss of function and improper membrane targeting [40]. When taken as a whole, these results consistently show that, in all species and experimental models, impaired membrane protein processing is a major cause of epithelial trafficking disease. Conserved abnormalities in epithelial transport pathways are further revealed by complementary functional studies in mice and humans [30]. Furthermore, abnormal protein processing directly impairs epithelial function, as demonstrated by decreased CFTR trafficking to the apical membrane [16]. This mechanism is also supported by human genetic mutation investigations, which demonstrate that changes in epithelial transport proteins lead to loss of function and improper membrane targeting [40]. When taken as a whole, these results consistently show that, in all species and experimental models, impaired membrane protein processing is a major cause of epithelial trafficking disease.

#### Impaired vesicular assembly and cargo sorting mechanism

Impaired intracellular cargo selection and trafficking fidelity are mostly caused by defects in vesicle coat machinery, which include disturbances of COPII/COPI systems, retromer function, and vesicle formation. More general disruptions in endosomal sorting machinery highlight the crucial role of coat-dependent regulation in intracellular trafficking [57]. Experimental evidence shows that dysfunction of the retromer complex leads to defective endosomal sorting and altered cargo recycling [54]. Sorting nexins are crucial for vesicular assembly and cargo sorting since they have also been demonstrated to promote selective cargo recognition and membrane modification during vesicle formation [47]. Furthermore, the intricacy of vesicle construction and lipid cargo packaging in epithelial cells is revealed by proteomic characterisation of prechylomicron transport vesicles [63]. Additionally, ARFRP1-dependent pathways support appropriate membrane structure and vesicle trafficking, highlighting the function of regulatory small GTPases in coat-mediated transport mechanisms [25]. All of these results point to the crucial disruption of epithelial trafficking caused by abnormalities in vesicle coat machinery, which affect intracellular transport, vesicle formation, and cargo selection.

#### Disruption of Rab GTPase–regulated intracellular trafficking

Since Rab proteins control vesicle targeting, recycling, and membrane identity, Rab GTPase-mediated trafficking abnormalities are a fundamental mechanism driving epithelial polarity and intracellular transport disorders. In knockout mice, disruption of Rab8 reduces apical trafficking and epithelial polarity, highlighting its crucial function in polarised membrane delivery [50]. Similar to this, disruption of Rab11-dependent recycling pathways leads to impaired membrane protein recycling and epithelial structure. These pathways are essential for endosomal return trafficking [34, 51]. The significance of effector-mediated control within Rab signalling networks is highlighted by regulatory interactions involving the Rab11–FIP2 complex, which further improve trafficking specificity [51]. There have also been reports of changes in Rab11, Rab8, and associated vesicle populations, suggesting coordinated failure across several Rab-regulated trafficking modules [60, 61]. Rab-dependent transport pathways are often necessary for preserving membrane polarity and guaranteeing precise cargo delivery in epithelial systems [30]. Furthermore, in vivo research in Drosophila shows that Rab21 has a role in tissue architecture and intestinal epithelial trafficking, supporting the conserved function of Rab GTPases across species [41]. All of these results point to Rab GTPase malfunction as a primary cause of loss of polarity and abnormalities in epithelial trafficking in several experimental paradigms.

#### Defective SNARE-dependent vesicle docking and fusion

Dysregulation of the SNARE machinery and associated membrane fusion processes represents a critical mechanism underlying epithelial trafficking disorders, as SNARE proteins and their regulatory partners coordinate vesicle docking and fusion with target membranes. Mutations in STXBP2/MUNC18-2 disrupt SNARE complex assembly and reduce vesicle fusion efficiency, leading to impaired intracellular trafficking [55]. Consistently, functional defects in Munc18-2 further emphasize its essential role in regulating SNARE-mediated membrane fusion events [60, 61]. Syntaxin 3 has been identified as a key apical SNARE component required for epithelial protein targeting, and its disruption results in mislocalisation of membrane cargo and loss of polarity [10]. In addition, αSNAP-dependent mechanisms are required for SNARE complex disassembly and recycling,impairment of this process disrupts vesicle fusion cycles and compromises membrane trafficking homeostasis [35]. Collectively, these findings demonstrate that SNARE complex dysfunction disrupts epithelial vesicle fusion and contributes to widespread defects in protein sorting and maintenance of membrane polarity.

#### Dysregulation of cytoskeletal motor-driven transport and sorting complexes

Adaptor proteins, motor proteins, and sorting signals represented a major mechanistic category, highlighting their central role in maintaining enterocyte polarity and apical trafficking. Disruptions in motor proteins such as MYO1A, MYO1B, MYO5B, and MYO6 were consistently associated with impaired cargo transport to the apical membrane. For instance, loss of MYO5B function leads to severe trafficking defects and enteropathy phenotypes [7, 14], while the importance of myosin-dependent transport in microvillus organisation and vesicle movement has been emphasised [21, 22, 31]. Additionally, defects in trafficking machinery contribute to impaired microvillus assembly, further underscoring the structural consequences of disrupted sorting [12].

Adaptor and sorting-related proteins also play crucial roles in epithelial trafficking regulation. Endotubin-mediated sorting has been demonstrated in polarised epithelial transport pathways [11], while coordinated MYO1B/MYO5B and SNX27-associated pathways highlight the complexity of apical trafficking regulation [26]. More extensive disruptions in apical trafficking machinery have also been reported in epithelial systems [13, 53]. Together, these findings show that abnormalities in sorting signals, motor proteins, and adaptor proteins cause extensive disruptions in vesicle targeting, cargo delivery, and epithelial organization, ultimately contributing to enterocyte dysfunction and disease.

#### Compromised endosomal recycling and membrane retrieval pathways

Disruption of endosomal recycling pathways represents a key mechanistic category, underscoring their essential role in maintaining enterocyte membrane composition and polarity. Impaired Rab11-dependent recycling has been shown to lead to defective apical protein recycling and mislocalisation when Rab11 targeting and regulation are disrupted [51, 52]. In addition, defective recycling contributes to tight junction internalization, thereby compromising epithelial barrier integrity [38]. Broader trafficking abnormalities, including accumulation of aberrant recycling vesicles and impaired cargo transport dynamics, have also been reported [59–61]. The retromer complex plays a critical role in endosomal sorting and membrane retrieval, and its dysfunction disrupts cargo recycling pathways [54]. Similarly, defects in endosomal sorting machinery further compromise intracellular trafficking and cargo retrieval processes [57]. Collectively, these findings indicate that impairment of endosomal recycling pathways leads to mislocalisation of key membrane proteins, loss of epithelial polarity, and disruption of intestinal barrier integrity, thereby contributing significantly to enterocyte pathophysiology.

### Risk-of-Bias summary

None of the studies was rated overall as low risk of bias or high risk of bias; instead, all 36 studies were rated as having “some concerns” under the applied criteria. This uniform classification was driven predominantly by four domains: sequence generation, allocation concealment, blinding of caregivers/researchers, and of outcome assessors, which were rated as unclear across the evidence base, largely reflecting incomplete reporting of randomisation and blinding procedures in the animal and in vitro mechanistic studies that make up most of the included evidence, where such procedures are not always applicable or explicitly described. By contrast, baseline characteristics, random housing, completeness of outcome data, absence of selective outcome reporting, and other sources of bias were rated low risk across all studies. Because no study met the pre-specified threshold for high risk of bias, the planned sensitivity analysis excluding high-risk studies did not alter the pool of evidence or the conclusions of the synthesis, as no study was removed. Overall, the uniform “some concerns” rating reflects gaps in methodological reporting rather than confirmed methodological flaws. Confidence in the synthesised mechanistic conclusions is therefore judged to be moderate: the consistency of findings across independent human, animal, and in vitro systems (Table 2) supports the core conclusions of this review, but the incomplete reporting of randomisation and blinding procedures means that the precision of individual study effect estimates cannot be fully verified.

## Discussion

The present systematic review synthesised evidence from 36 studies on enterocyte trafficking proteins and the pathophysiological consequences of their sortopathies. The findings confirm that intracellular protein sorting is not merely a supporting process, but a central determinant of enterocyte function, and its disruption leads to a spectrum of inherited and acquired intestinal disorders.

### Enterocyte polarity and trafficking integration

The findings support a model in which enterocyte polarity is maintained not by isolated molecular pathways but by a single, hierarchically organized trafficking network. At the upstream regulatory tier, SAR1B-dependent COPII vesicles and ARFRP1-controlled Golgi lipidation govern biosynthetic cargo exit [25, 63]. The retromer-SNX27 complex retrieves internalized receptors and transporters from early endosomes back to the plasma membrane [54, 57], and the Rab GTPase hub, principally Rab8a and Rab11a, confers vesicle identity and directionality [41, 50, 52]. These upstream inputs are interdependent: Rab11a recruits retromer-associated effectors, so retromer failure can secondarily impair Rab11-dependent recycling [29]. At the effector tier, the Rab11–FIP2–MYO5B axis converts Rab-GTP identity into actin-based apical movement [51], a coordinated myosin ensemble (MYO5B, MYO1B, MYO1A, MYO6) controls vesicle directionality along the brush border cytoskeleton [14, 21, 26, 31], and the SNARE complex Syntaxin 3, MUNC18-2, and αSNAP executes terminal membrane fusion licensed by Rab8a-GTP [10, 35, 60, 61]. Disruption at any single node, therefore, creates a shared bottleneck: vesicles accumulate subapically, unable to complete apical delivery.

Regardless of which component is mutated, network dysfunction converges on three overlapping pathological outputs: defective apical delivery (MYO5B/SAR1B loss), defective endocytic retrieval (SNX27/retromer failure), and defective exocytic fusion (STXBP2/SNARE blockade), each sufficient to cause severe enteropathy, as seen in MVID, chylomicron retention disease, and FHL5 enteropathy, respectively [40, 55]. These outputs are not mutually exclusive: MYO5B deficiency simultaneously impairs apical delivery and Rab11a-dependent recycling, compounding disease severity. The network also exhibits a self-reinforcing vulnerability: trafficking failure erodes polarity cues, as evidenced by MYO5B deficiency impairing Par3/aPKC recruitment [14], and polarity loss, in turn, disrupts the spatial reference that trafficking requires, creating a feedback loop that accelerates clinical deterioration. Enterocyte polarity and the trafficking network are therefore inextricably co-dependent: the network builds and sustains polarity, while polarity provides the coordinates on which directed trafficking depends.

### Mechanisms linking defects to disease

The three mechanistic pathways described below, defective apical delivery, defective endocytic retrieval, and defective exocytic fusion, should not be understood as independent disease mechanisms but as three failure modes of the single trafficking network described in Sect. "Enterocyte polarity and trafficking integration". Under normal conditions, this network establishes and maintains epithelial polarity by continuously delivering apical proteins to the brush border, retrieving internalised proteins from early endosomes, and executing terminal vesicle fusion to sustain the apical membrane composition. Polarity is therefore not a fixed structural property but a dynamic steady state actively maintained by the trafficking network, and when any node is disrupted, whether Rab GTPase identity, motor-driven transport, SNARE-mediated fusion, or retromer-dependent retrieval, this steady state collapses, with apical proteins accumulating subapically, tight junctions internalising, and absorptive function lost [14, 38, 40]. It is this collapse, rather than the loss of any single transporter, that constitutes the shared pathological mechanism of sortopathies and explains why mutations in structurally unrelated proteins MYO5B, SAR1B, and STXBP2 converge on a clinically similar phenotype of severe early-onset enteropathy.

Newly synthesised or recycled transporters cannot reach the brush border due to defective apical delivery. MYO5B mutations in microvillus inclusion disease (MVID), where Rab11–Rab8–positive recycling endosomes build up and are unable to fuse with the apical membrane, served as an example of this process [14, 60, 61]. Similar to this, SAR1B mutations in chylomicron retention disease prevent lipid-laden chylomicrons from leaving the endoplasmic reticulum by blocking the pre-chylomicron transport vesicle (PCTV) [63]. Loss of absorptive function results from the inability of nutrients and electrolytes to reach the luminal surface in both situations. Recycling endosome arrest (Rab11a, MYO5B) and ER-to-Golgi transport failure (SAR1B, ARFRP1) are important sub-mechanisms within this route.

Transporters are kept on the plasma membrane due to defective endocytic retrieval, which interferes with normal turnover, signalling, and lipid balance. Because internalization was hampered, MYO6 loss caused a buildup of NHE3 and NaPi2b near the brush boundary, as observed in Snell's waltzer mice [21]. This paradoxically raises some transporters' surface expression while blocking their controlled recycling, which could lead to improper ion retention. Impaired clathrin-mediated endocytosis (MYO6, caveolin-1 pathways) and cargo sorting into recycling endosomes (SNX27, retromer defects) are examples of sub-mechanisms.

Cargo-laden vesicles accumulate under the brush boundary as a result of defective exocytic fusion, which prevents vesicles from being fully inserted into the apical membrane. Even though intracellular CFTR levels were normal, syntaxin 3 impairment prevented the channel from reaching the surface by blocking cAMP- and cGMP-stimulated CFTR exocytosis [10, 16]. Similarly, a fusion blockade at the apical plasma membrane was indicated by the accumulation of syntaxin 3-positive vesicles and microvillus shortening caused by STXBP2 (MUNC18-2) mutations in FHL5 enteropathy [55, 61]. Sub-mechanisms include Munc18-2 regulation malfunction (STXBP2) and SNARE complex assembly failure (syntaxin 3, SNAP25, VAMP8). Importantly, these three pathways are not mutually exclusive,MYO5B deficiency impairs both apical delivery and aspects of endosomal recycling, creating a compounded defect that likely explains the severity of MVID.

### Comparative analysis of sortopathies

Comparing the three well‑characterised genetic sortopathies, MVID, chylomicron retention disease, and FHL5, enteropathy reveals both commonalities and distinctions. Common features include: (i) all are caused by mutations in genes encoding non‑redundant trafficking proteins (MYO5B, SAR1B, STXBP2); (ii) all present with severe, early‑onset diarrhoea and failure to thrive; (iii) all show ultrastructural evidence of vesicle accumulation (subapical vesicles in MVID and FHL5, ER‑retained chylomicrons in Anderson disease); and (iv) all involve mislocalisation of apical cargo (NHE3/CFTR in MVID, ApoB‑containing lipoproteins in chylomicron retention disease, syntaxin 3‑positive vesicles in FHL5). Distinctive features relate to the specific cargo affected: MVID primarily disrupts ion transporters (NHE3, CFTR), causing electrolyte‑driven watery diarrhoea; chylomicron retention disease selectively impairs lipid transport, causing fat malabsorption without significant electrolyte loss; FHL5 enteropathy combines trafficking defects with systemic immune dysregulation (hemophagocytic lymphohistiocytosis), indicating that STXBP2 has broader cell‑type functions beyond the intestine. Interestingly, Rab8a and Rab11a knockout mice phenocopy aspects of MVID [50, 52], yet human mutations in these Rabs have not been identified, suggesting either embryonic lethality or that redundant mechanisms exist in humans. This comparative analysis supports the concept that sortopathies are defined not only by the trafficking protein involved but also by the cargoes that depend on that protein for correct localisation.

### Diagnostic and therapeutic implications

The findings have several translational implications. Diagnostically, the consistent association of MYO5B mutations with MVID and SAR1B mutations with chylomicron retention disease supports targeted genetic testing in infants with congenital diarrhoea. However, the observation that some MVID patients lack MYO5B mutations [40] suggests that other trafficking genes, including Rab8a, Rab11a, or syntaxin 3, may be candidate genes for unsolved cases. Electron microscopy remains the gold standard for diagnosing MVID (microvilli inclusions) and chylomicron retention disease (lipid droplets in enterocytes), but our review highlights that immunostaining for mislocalised transporters (e.g., NHE3, CFTR) could serve as a rapid screening tool.

Therapeutically, the current management of sortopathies is largely supportive (parenteral nutrition, fat‑restricted diet). However, the elucidation of molecular mechanisms opens avenues for targeted interventions. Pharmacological chaperones that stabilise misfolded trafficking proteins have been successful in other disorders (e.g., cystic fibrosis) but have not been tested in MVID. Gene therapy is conceptually attractive for MYO5B, SAR1B, or STXBP2 deficiencies, but the large size of MYO5B (over 6,000 amino acids) poses delivery challenges. Small molecules that modulate alternative trafficking pathways, for example, enhancing Rab11a activity or upregulating compensatory myosins, could be explored. The finding that mTORC1 regulates PCTV formation [39] suggests that mTOR inhibitors might paradoxically worsen chylomicron retention, whereas activators could be beneficial, but this requires careful testing. Finally, antisense oligonucleotides or CRISPR‑based editing could correct specific mutations, but these approaches remain preclinical.

### Research gaps and future directions

Despite significant progress, several gaps remain. First, most studies relied on animal models or cell lines; human tissue data are limited to small case series. Large‑scale, prospective studies of patients with unexplained congenital diarrhoea are needed to identify novel trafficking gene mutations. Second, the role of acquired trafficking defects in common intestinal diseases (inflammatory bowel disease, infectious diarrhoea, coeliac disease) is underexplored. The observation that TNF‑α induces occludin endocytosis [38] suggests that inflammatory cytokines may broadly disrupt enterocyte trafficking, but whether this contributes to disease progression or represents a therapeutic target is unknown. Third, compensatory mechanisms in trafficking networks are poorly understood. The finding that Myo1a/Myo6 double knockout restores some membrane tethering defects (Hegan et al., 2012b) indicates that enterocytes can adapt, but the molecular basis of this compensation remains unclear. Fourth, high‑throughput screening for small molecules that restore trafficking in patient‑derived organoids (e.g., MVID enteroids) could identify lead compounds for drug repurposing. Fifth, the interface between trafficking and metabolism – particularly how nutrient availability modulates trafficking protein expression has received little attention. Given that Rab11a and SNX27 are involved in glucose and metal ion transport [54], bidirectional crosstalk between nutritional status and trafficking efficiency is plausible. Finally, the development of non‑invasive biomarkers of trafficking dysfunction (e.g., faecal exosomal cargo, serum autoantibodies against mislocalised transporters) could facilitate diagnosis and monitoring of sortopathies without repeated biopsies. A further methodological limitation is the absence of prospective protocol registration,however, given the mechanistic and experimental nature of this review, which did not involve pooling of clinical intervention or patient outcome data, the risk of selective outcome reporting that registration is designed to prevent was considered low. Restricting the search to studies published from 2000 onward may have excluded earlier foundational investigations of intracellular trafficking proteins. However, these earlier studies mainly established the basic biology of trafficking molecules, whereas the review was designed to synthesise contemporary mechanistic evidence relevant to enterocyte function and intestinal disease.

## Conclusions and recommendations

This systematic review establishes that enterocyte trafficking proteins Rab GTPases, myosin motors, SNAREs, and retromer components are essential for maintaining apical polarity, localising transporters, and enabling nutrient absorption. Their dysfunction underlies a group of severe disorders collectively termed sortopathies, including microvillus inclusion disease, chylomicron retention disease, and FHL5‑associated enteropathy. Mechanistically, defective apical delivery, impaired endocytic retrieval, and blocked exocytic fusion converge on common pathways of transporter mislocalisation and barrier breakdown. Future research should focus on identifying novel sortopathy genes, exploring acquired trafficking defects in inflammatory bowel disease, and developing targeted therapies that restore normal protein sorting.

Based on the findings of this systematic review, we recommend that genetic testing for MYO5B, SAR1B, and STXBP2 be included in first‑line diagnostic panels for infants with congenital diarrhoea of unknown aetiology, with electron microscopy and immunostaining for NHE3 and CFTR serving as adjunctive diagnostic tools. Multidisciplinary care involving paediatric gastroenterologists, clinical geneticists, dietitians, and pathologists is essential for managing sortopathy patients. Research priorities should include establishing patient registries and biobanks, developing patient‑derived enteroid models for high‑throughput drug screening, and investigating acquired trafficking defects in common inflammatory conditions. Funding agencies and international collaborations are needed to accelerate preclinical development of AAV‑based gene therapy for MYO5B, evaluation of mTORC1 modulators in chylomicron retention disease, and testing of pharmacological chaperones in MYO5B mutant cell lines. Ultimately, these efforts aim to translate mechanistic insights into targeted therapies for patients suffering from these devastating intestinal disorders.

## Supplementary Information

Supplementary Material 1.

Supplementary Material 2.

Supplementary Material 3.

Supplementary Material 4.

## Data Availability

The Supplementary materials for this article are submitted alongside the manuscript and contain the data analysed in this review. Supplementary File S1: PRISMA checklist 2020; Supplementary File S2: Search Strings; Supplementary File S3: Data extraction sheet; Supplementary File S4: Risk of Bias assessment report.
