# Supplementary material for: Enterocyte trafficking proteins and the pathophysiological consequences of their sortopathies
Source: BMC Gastroenterol. 2026 Aug 12;26:746. doi: 10.1186/s12876-026-05205-6 (PMC13749957; doi:10.1186/s12876-026-05205-6)
Supplement: Supplementary file 1 [file 12876_2026_5205_MOESM1_ESM.docx]

| **Section and Topic** | **Item #** | **Checklist item** | **Location where item is reported** |
| --- | --- | --- | --- |
| **TITLE** | | |  |
| Title | 1 | Identify the report as a systematic review. | **Enterocyte Trafficking Proteins and the Pathophysiological Consequences of Their Sortopathies** |
| **ABSTRACT** | | |  |
| Abstract | 2 | See the PRISMA 2020 for Abstracts checklist. | **Background:** Enterocytes rely on highly coordinated intracellular trafficking systems to maintain apical-basolateral polarity, enabling efficient nutrient absorption, ion transport, and barrier integrity. Disruption of protein sorting pathways has emerged as a key mechanism underlying a group of intestinal disorders termed enterocyte sortopathies. However, the molecular basis linking trafficking defects to intestinal pathophysiology remains incompletely understood  **Objectives:** This systematic review aimed to synthesize current evidence on enterocyte trafficking proteins and to elucidate the molecular mechanisms by which their dysfunction contributes to intestinal disease  **Methods:** A systematic literature search was conducted in PubMed/MEDLINE, Web of Science, and Wiley Online Library for studies published between January 2000 and March 2026, following PRISMA 2020 guidelines. Studies involving enterocyte trafficking proteins, including Rab GTPases, myosin, SNAREs, and sorting nexins, were included based on predefined PICO criteria. Data were extracted and analyzed using mechanistic categorization and thematic synthesis.  **Results:** A total of 36 studies were included, identifying key trafficking regulators, including Rab8a, Rab11a, MYO5B, MYO1A, MYO6, syntaxin 3, MUNC18-2, and SNX27, as essential for enterocyte protein sorting and polarity. Six major mechanisms of dysfunction were observed, involving defects in protein maturation, vesicle formation, Rab-mediated trafficking, SNARE-dependent fusion, motor-driven transport, and endosomal recycling. These disruptions converge into three main pathological processes: impaired apical delivery, defective endocytic retrieval, and failed exocytic fusion, ultimately leading to severe congenital enteropathies such as microvillus inclusion disease, chylomicron retention disease, and STXBP2-associated enteropathy.  **Conclusion:** Enterocyte trafficking proteins are fundamental determinants of epithelial polarity and intestinal function. Their dysfunction drives a spectrum of severe intestinal disorders through converging mechanisms of protein mislocalization and barrier disruption. Advancing diagnostic approaches and developing targeted therapies aimed at restoring intracellular trafficking pathways represent critical priorities for improving patient outcomes  **Keywords:** Enterocyte trafficking, Protein sorting, Sortopathies, Trafficking proteins. |
| **INTRODUCTION** | | |  |
| Rationale | 3 | Describe the rationale for the review in the context of existing knowledge. | Section 1. Introduction |
| Objectives | 4 | Provide an explicit statement of the objective(s) or question(s) the review addresses. | Section 1. Introduction |
| **METHODS** | | |  |
| Eligibility criteria | 5 | Specify the inclusion and exclusion criteria for the review and how studies were grouped for the syntheses. | Section 2.3 Eligibility criteria guided by the PICO framework  Section 2.5 Mechanistic Categorization and Thematic Analysis |
| Information sources | 6 | Specify all databases, registers, websites, organizations, reference lists, and other sources searched or consulted to identify studies. Specify the date when each source was last searched or consulted. | Section 2.2 Search strategy |
| Search strategy | 7 | Present the full search strategies for all databases, registers, and websites, including any filters and limits used. | Section 2.2 Search strategy and supplementary file S2 |
| Selection process | 8 | Specify the methods used to decide whether a study met the inclusion criteria of the review, including how many reviewers screened each record and each report retrieved, whether they worked independently, and if applicable, details of automation tools used in the process. | Section 2.4 Selection, Data Extraction, and Analysis, and Supplementary file S3 |
| Data collection process | 9 | Specify the methods used to collect data from reports, including how many reviewers collected data from each report, whether they worked independently, any processes for obtaining or confirming data from study investigators, and, if applicable, details of automation tools used in the process. | Section 2.4 Selection, Data Extraction, and Analysis, and Supplementary file S3 |
| Data items | 10a | List and define all outcomes for which data were sought. Specify whether all results that were compatible with each outcome domain in each study were sought (e.g. for all measures, time points, analyses), and if not, the methods used to decide which results to collect. | **2**.5. Mechanistic Categorization and Thematic Analysis |
|  | 10b | List and define all other variables for which data were sought (e.g. participant and intervention characteristics, funding sources). Describe any assumptions made about any missing or unclear information. | Section 2.4 Selection, Data Extraction, and Analysis, and Supplementary file S3 |
| Study risk of bias assessment | 11 | Specify the methods used to assess risk of bias in the included studies, including details of the tool(s) used, how many reviewers assessed each study and whether they worked independently, and if applicable, details of automation tools used in the process. | 2.6. Risk of Bias (RoB) Assessment |
| Effect measures | 12 | Specify for each outcome the effect measure(s) (e.g. risk ratio, mean difference) used in the synthesis or presentation of results. | NA |
| Synthesis methods | 13a | Describe the processes used to decide which studies were eligible for each synthesis (e.g., tabulating the study intervention characteristics and comparing against the planned groups for each synthesis (item #5)). | Section 2.4 Selection, Data Extraction, and Analysis, and Supplementary file S3 |
|  | 13b | Describe any methods required to prepare the data for presentation or synthesis, such as handling of missing summary statistics, or data conversions. | NA |
|  | 13c | Describe any methods used to tabulate or visually display results of individual studies and syntheses. | Table2: Mechanism-Specific Roles of Enterocyte Trafficking Proteins in Sortopathies |
|  | 13d | Describe any methods used to synthesize results and provide a rationale for the choice(s). If meta-analysis was performed, describe the model(s), method(s) to identify the presence and extent of statistical heterogeneity, and software package(s) used. | NA |
|  | 13e | Describe any methods used to explore possible causes of heterogeneity among study results (e.g. subgroup analysis, meta-regression). | NA |
|  | 13f | Describe any sensitivity analyses conducted to assess robustness of the synthesized results. | 2.5. Mechanistic Categorization and Thematic Analysis |
| Reporting bias assessment | 14 | Describe any methods used to assess risk of bias due to missing results in a synthesis (arising from reporting biases). | Section 2.6 Risk of bias assessment |
| Certainty assessment | 15 | Describe any methods used to assess certainty (or confidence) in the body of evidence for an outcome. | Section 2.6 Risk of bias assessment |
| **RESULTS** | | |  |
| Study selection | 16a | Describe the results of the search and selection process, from the number of records identified in the search to the number of studies included in the review, ideally using a flow diagram. |  |
|  | 16b | Cite studies that might appear to meet the inclusion criteria, but which were excluded, and explain why they were excluded. | NA |
| Study characteristics | 17 | Cite each included study and present its characteristics. | Table 2: Mechanism-Specific Roles of Enterocyte Trafficking Proteins in Sortopathies |
| Risk of bias in studies | 18 | Present assessments of risk of bias for each included study. | Section 2.6 Risk of Assessment and Supplementary file S4 |
| Results of individual studies | 19 | For all outcomes, present, for each study: (a) summary statistics for each group (where appropriate) and (b) an effect estimate and its precision (e.g. confidence/credible interval), ideally using structured tables or plots. | NA |
| Results of syntheses | 20a | For each synthesis, briefly summarise the characteristics and risk of bias among contributing studies. | Sections 3.1, 3.2, 3.3, 3.4 ,3.5and Supplementary file S3 |
|  | 20b | Present results of all statistical syntheses conducted. If meta-analysis was done, present for each the summary estimate and its precision (e.g. confidence/credible interval) and measures of statistical heterogeneity. If comparing groups, describe the direction of the effect. | NA |
|  | 20c | Present results of all investigations of possible causes of heterogeneity among study results. | NA |
|  | 20d | Present results of all sensitivity analyses conducted to assess the robustness of the synthesized results. | NA |
| Reporting biases | 21 | Present assessments of risk of bias due to missing results (arising from reporting biases) for each synthesis assessed. | 2.6. Risk of Bias (RoB) Assessment and Supplementary File S4 |
| Certainty of evidence | 22 | Present assessments of certainty (or confidence) in the body of evidence for each outcome assessed. | 2.6. Risk of Bias (RoB) Assessment and Supplementary File S4 |
| **DISCUSSION** | | |  |
| Discussion | 23a | Provide a general interpretation of the results in the context of other evidence. | Section 3. Result  Enterocyte trafficking disorders arise from failures in protein processing, vesicle formation, Rab- and SNARE-mediated transport, cytoskeletal motor function, and endosomal recycling-leading to mislocalized membrane proteins, loss of polarity, and impaired epithelial function. |
|  | 23b | Discuss any limitations of the evidence included in the review. | 4.5 Research Gaps and Future Directions |
|  | 23c | Discuss any limitations of the review processes used. | NA |
|  | 23d | Discuss implications of the results for practice, policy, and future research. | 4.4. Diagnostic and Therapeutic Implications  4.5 Research Gaps and Future Directions |
| **OTHER INFORMATION** | | |  |
| Registration and protocol | 24a | Provide registration information for the review, including register name and registration number, or state that the review was not registered. | This review was not prospectively registered. |
|  | 24b | Indicate where the review protocol can be accessed, or state that a protocol was not prepared. | There was no established protocol |
|  | 24c | Describe and explain any amendments to information provided at registration or in the protocol. | There was no established protocol and review was not prospectively registered. |
| Support | 25 | Describe sources of financial or non-financial support for the review, and the role of the funders or sponsors in the review. | This review did not receive any special funding. |
| Competing interests | 26 | Declare any competing interests of review authors. | No conflicting interests are disclosed by the author or authors. |
| Availability of data, code and other materials | 27 | Report which of the following are publicly available and where they can be found: template data collection forms; data extracted from included studies; data used for all analyses; analytic code; any other materials used in the review. | The author provided the data extraction sheet, study characteristics table, and supporting review materials. Where appropriate, supplemental materials are included in the appendices. |

*From:*  Page MJ, McKenzie JE, Bossuyt PM, Boutron I, Hoffmann TC, Mulrow CD, et al. The PRISMA 2020 statement: an updated guideline for reporting systematic reviews. BMJ 2021;372:n71. doi: 10.1136/bmj.n71. This work is licensed under CC BY 4.0. To view a copy of this license, visit <https://creativecommons.org/licenses/by/4.0/>
