# Supplementary material for: Enterocyte trafficking proteins and the pathophysiological consequences of their sortopathies
Source: BMC Gastroenterol. 2026 Aug 12;26:746. doi: 10.1186/s12876-026-05205-6 (PMC13749957; doi:10.1186/s12876-026-05205-6)
Supplement: Supplementary file 2 [file 12876_2026_5205_MOESM2_ESM.docx]

**Supplementary file S2:Search strategies**

A systematic literature search was conducted to identify studies on enterocyte trafficking proteins and their pathophysiological consequences. Relevant articles were retrieved from major databases including PubMed, Web of Science, and Wiley Online Library. The search combined controlled vocabulary and free-text terms related to enterocyte biology, protein trafficking, and key regulatory proteins such as Rab GTPases, SNAREs, myosins, sorting nexins, and retromer complexes, using Boolean operators to ensure appropriate sensitivity and specificity.

**Search strategy parameters:**

- Time frame: From database inception to the most recent available literature
- Language: English
- Search fields: Title, abstract, and keywords (where applicable)
- Boolean operators: AND, OR were used to combine search terms
- Truncation/wildcards: Applied (e.g., enterocyte*, trafficking*) to capture word variations

**1.PubMed**

(enterocyte* OR "intestinal epithelial cell*" OR epithelium)

AND

("protein trafficking" OR "membrane trafficking" OR "vesicular transport" OR "protein transport")

AND

(Rab OR SNARE OR myosin OR "sorting nexin*" OR retromer OR trafficking)

**2.Wiley online Library**

(enterocyte* OR "intestinal epithelial cell*")

AND

("membrane trafficking" OR "protein trafficking")

AND

(Rab OR SNARE OR myosin OR retromer)

**3.Web of Science**

TS=(

(enterocyte* OR "intestinal epithelial cell*")

AND

("protein trafficking" OR "membrane trafficking")

AND

(Rab OR SNARE OR myosin OR "sorting nexin*" OR retromer)

AND

(polarity OR "apical trafficking")

AND

(disease OR mutation* OR enteropathy))
